# Genomic Landscape of a Three-Generation Pedigree Segregating Affective Disorder

**DOI:** 10.1371/journal.pone.0004474

**Published:** 2009-02-13

**Authors:** Shuzhang Yang, Kai Wang, Brittany Gregory, Wade Berrettini, Li-San Wang, Hakon Hakonarson, Maja Bucan

**Affiliations:** 1 Department of Genetics, University of Pennsylvania, Philadelphia, Pennsylvania, United States of America; 2 Department of Psychiatry, University of Pennsylvania, Philadelphia, Pennsylvania, United States of America; 3 Pathology and Laboratory Medicine, University of Pennsylvania, Philadelphia, Pennsylvania, United States of America; 4 Penn Center for Bioinformatics, University of Pennsylvania, Philadelphia, Pennsylvania, United States of America; 5 Center for Applied Genomics, The Children's Hospital of Philadelphia, Philadelphia, Pennsylvania, United States of America; 6 Division of Genetics, The Children's Hospital of Philadelphia, Philadelphia, Pennsylvania, United States of America; James Cook University, Australia

## Abstract

Bipolar disorder (BPD) is a common psychiatric illness with a complex mode of inheritance. Besides traditional linkage and association studies, which require large sample sizes, analysis of common and rare chromosomal copy number variants (CNVs) in extended families may provide novel insights into the genetic susceptibility of complex disorders. Using the Illumina HumanHap550 BeadChip with over 550,000 SNP markers, we genotyped 46 individuals in a three-generation Old Order Amish pedigree with 19 affected (16 BPD and three major depression) and 27 unaffected subjects. Using the PennCNV algorithm, we identified 50 CNV regions that ranged in size from 12 to 885 kb and encompassed at least 10 single nucleotide polymorphisms (SNPs). Of 19 well characterized CNV regions that were available for combined genotype-expression analysis 11 (58%) were associated with expression changes of genes within, partially within or near these CNV regions in fibroblasts or lymphoblastoid cell lines at a nominal P value <0.05. To further investigate the mode of inheritance of CNVs in the large pedigree, we analyzed a set of four CNVs, located at 6q27, 9q21.11, 12p13.31 and 15q11, all of which were enriched in subjects with affective disorders. We additionally show that these variants affect the expression of neuronal genes within or near the rearrangement. Our analysis suggests that family based studies of the combined effect of common and rare CNVs at many loci may represent a useful approach in the genetic analysis of disease susceptibility of mental disorders.

## Introduction

Recent large-scale studies showed a high degree of copy number variation (CNV) in the human genome, suggesting that CNVs may account for a significant proportion of human phenotypic variation and disease susceptibility [1]–[6]. A significant fraction of CNVs are likely to have functional consequences due to gene dosage alteration, disruption of genes or gene-fusion, positional effects, or the uncovering of deleterious alleles [7]. Genome-wide searches for CNVs associating with schizophrenia identified a greater burden of structural variation in individuals with schizophrenia than in control subjects [8]–[11]. Moreover, associations with schizophrenia were found for large deletions at 1q21, 15q11.2 and 15q13.3 [11]. These studies support the idea that many loci may contribute to the disease and that these genetic factors may be common for several neuropsychiatric disorders.

The Old Order Amish is a genetically isolated population of European descent located predominantly in Central Pennsylvania [12], with large families segregating mental illness as well as several metabolic and neurological disorders [13]–[16]. The advantages of studying mental illness in the Old Order Amish, among others, include: (1) The families are geographically and genetically isolated, with a potentially reduced number of risk-factors for a disease compared to a more heterogeneous population; (2) Large sibships allow more direct comparisons between affected and unaffected individuals in the same family; (3) Similar environmental influences, including lack of alcohol and drug use, may minimize the potential confounding factors that contribute to disease susceptibility [17]. The neuropsychiatric genetic studies in Old Order Amish pedigrees included analysis of major affective disorders (bipolar and unipolar forms). The original genetic linkage studies in this pedigree reported positive findings on chromosome 11 (11p15) [18]. However, a re-evaluation of extended pedigrees and clinical updates did not support the original finding [19]–[21]. Subsequent genome-wide linkage analysis using 551 microsatellite markers revealed a complex mode of inheritance with possible susceptibility loci on chromosomes 6, 13 and 15 [22] and a protective locus on 4p15 [23].

In this study, we used high density SNP genotype data to identify structural variants in the core Old Order Amish pedigree and two extensions (Coriell Institute for Medical Research cell repository family number 884) segregating mood or affective disorders (BPD and major depression). We explored the potential functional consequence of these genomic variants by examining their frequency, size and gene content in affected and unaffected family members, and their effects on gene expression in fibroblast and/or lymphoblastoid cell-lines (LCLs). Our results indicate presence of multiple micro-deletions and micro-duplications, segregating in the large pedigree. Although the average number and size of CNVs do not differ in affected and unaffected individuals, we show that 58% of the tested CNVs (11 out of 19) were associated with expression changes of genes within, partially within or near these CNV regions in fibroblasts or LCLs. Several CNVs frequently found in the affected family members alter expression levels of genes involved in neurological functions. Our results reveal previously unrecognized complex patterns of inheritance for groups of CNVs.

## Results

### Genotyping and CNV identification

The three-generation Old Order Amish family 884 consists of 51 individuals, including 32 clinically unaffected family members and 19 family members with affective disorders; among the 19 affected subjects, 16 have bipolar disorder type I (BPI), type II (BPII) or not otherwise specified (BP-NOS), three with major depression (MDD (Supplemental Table S1). Apart from general medical histories abstracted for all patients with psychiatric medical records (often multiple admissions) no additional general medical screening for non-psychiatric conditions was done. However, none of the important metabolic or neurological disorders commonly found in the Amish were mentioned in their psychiatric medical records and/or observed during decades of contact with the subjects used in this study ( J. A. Egeland and A. M. Hostetter, personal communication; see below).

DNA samples isolated from fibroblasts and/or lymphoblastoid cell lines (when fibroblasts were not available) from these 51 individuals were genotyped with the Illumina HumanHap550 SNP genotyping array; 46 samples gave high quality data that were subjected to CNV analysis. The five subjects with failed genotyping include two healthy unaffected and three individuals with a minor depressive disorder.

A non-parametric SNP-based linkage analysis performed using the Merlin program [24] did not give significant or suggestive linkage signals (maximum LOD score of 1.62 for chr14: 27.526–29.525 Mb), further suggesting a complex mode of inheritance with possible multiple low risk susceptibility loci, rather than a risk attributable to a major gene(s). Based on this finding and recent insights into the role of structural variants in etiology of neuropsychiatric diseases, we attempted to utilize these high-density SNP-genotype data to assess the extent of structural variation in this family and to examine the effect of CNVs on the expression of genes within and near breakpoints.

**Figure 1:**
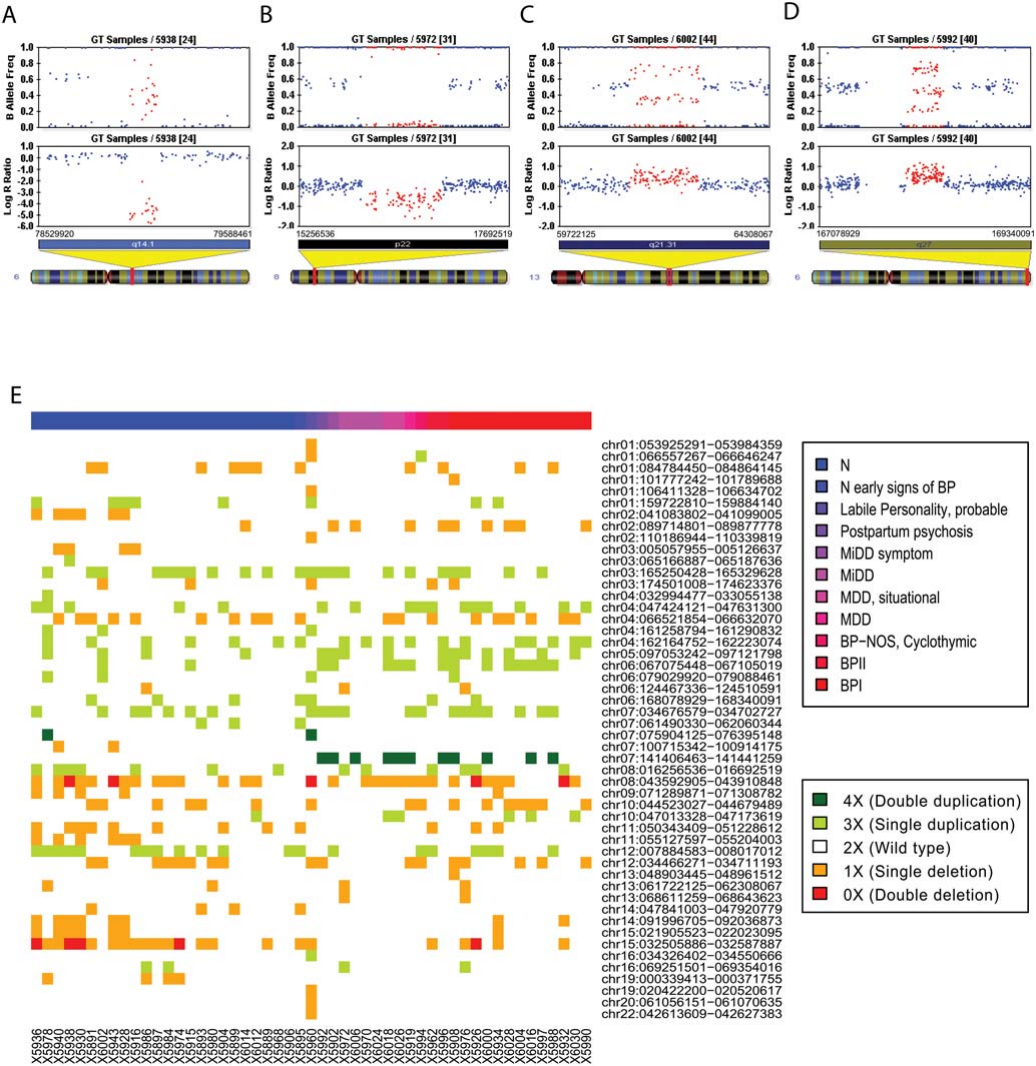
Characteristic signal patterns of copy number states and the distribution of CNVs in family 884. (A–D) Visualization of signal intensity for the CNV regions with 0 copy (A), 1 copy (B), 3 copies (C) and 4 copies (D) in the Illumina BeadStudio software. For each SNP, the Log R Ratio (LRR) is a normalized measure of signal intensity for two alleles of a SNP, while the B Allele Frequency (BAF) is a normalized measure of the allelic intensity ratio of the two alleles. Different copy number states have characteristic patterns of LRR and BAF. (E) Visualization of the CNVs by heatmap. CNVs are ordered by chromosomes then by chromosomal positions by the NCBI Release 36 Human Genome. Clinical status for all subjects is indicated by color; the respective hierarchical clustering was then computed for each group.

We used a high-resolution CNV detection algorithm, PennCNV [25], to call CNVs from the signal intensity data, with a threshold of 10 SNPs. This threshold was previously demonstrated to result in a low false positive rate for high-quality samples [25], [26]. The PennCNV algorithm allowed us to detect four abnormal copy number states other than the normal diploid state: deletion by one or two copies and duplication by one or two copies (Figure 1A). In total, we identified 388 CNVs that were classified to 50 unique CNV regions (Table 1). Twenty-three of these CNV regions map to genic and 27 CNVs to intergenic regions (Table 1). The distribution of the CNVs present in four or more subjects are illustrated in Supplemental Figure S1. The number of CNVs ranges from four to 20 for each individual (mean = 8.5; SD = 2.9). The size of these CNVs ranges from 12,447 bp to 885,204 bp (mean = 148,135 bp, median = 80,890 bp). The average number and size of CNVs that exist in affected individuals (including BPD and major depression) are not significantly different from those of CNVs exist in unaffected individuals (*P* = 0.300 and 0.237 respectively, t-test), nor are they significantly different between BPD and unaffected individuals (*P* = 0.321 and 0.187 respectively, t-test). The large pedigree size precludes the use of standard family-based association test, we instead performed a permutation procedure to adjust for the sibship relationships (Supplemental Text S1). Although the permutation test revealed no significant association between CNV genotype and disease status (Supplemental Table S2 and S3), we found four CNV regions (on 6q27, 9q21.11, 12p13.31 and 15q11.2.) at a nominally higher frequency in individuals with affective disorders in comparison to healthy family members.

**Table 1:** Fifty CNV regions identified in Amish family 884.

CNV region^1^	#SNP	Length (Kb)	CN^2^	Common or rare^3^	#subjects (#affected)^4^	Contained Genes	Partially contained genes	Previously reported linkage Marker^5^
chr1:53925291–53984359	14	59069	1	rare^3^	1(0)		GLIS1	
chr1:66557267–66646247	29	88981	1	rare^3^	1(0)		PDE4B	
chr1:84784450–84864145	16	79696	1	rare^3^	1(0)	CTBS	SPATA1	
chr1:101777242–101789688	10	12447	1	rare	4(0)			
chr1:106411328–106634702	37	223375	3	rare	4(1)			
chr1:159722810–159884140	15	161331	3	common	1(0)	FCGR2A,FCGR2C,FCGR3A,FCGR3B,HSPA6		D1S2675
chr2:41083802–41099005	10	15204	1&0	common	23(5)			
chr2:89714801–89877778	10	162978	1	common	7(1)			
chr2:110186944–110339819	15	152876	1	common	8(2)	MALL,NPHP1		
chr3:5057955–5126637	24	68683	1	rare^3^	4(0)			
chr3:65166887–65187636	11	20,750	1	common	2(1)			
chr3:165250428–165329628	10	79201	1	rare	4(1)			
chr3:174501008–174623376	22	122369	1	rare^3^	1(1)		NLGN1 long isoform	D3S1565
chr4:32994477–33055138	15	60662	1	rare	17(7)			
chr4:47424121–47631300	23	207180	3	rare^3^	20(6)	NFXL1	CORIN	
chr4:66521854–66632070	11	110217	1	rare^3^	6(0)			
chr4:161258794–161290832	20	32039	1	common	12(3)			
chr4:162164752–162223074	14	58323	3	common	6(5)			
chr5:97053242–97121798	15	68557	1	common	16(9)			
chr6:67075448–67105019	13	29572	1	common	5(1)			
chr6:79029920–79088461	24	58542	1&0	common	30(12)			
chr6:124467336–124510591	12	43256	3	common	13(4)		NKAIN	not reported
chr6:168078929–168340091	120	261163	4	common	11(8)	FRMD1,KIF25	MLLT4	
chr7:34676579–34702727	12	26149	1	rare	2(0)		NPSR1	
chr7:61490330–62060344	17	570015	4	common	2(0)			
chr7:75904125–76395148	23	491024	3	rare	3(0)	DTX2,POMZP3,UPK3B	ZP3	
chr7:100715342–100914175	35	198834	3	rare	20(9)	RABL5	EMID2	D7S518
chr7:141406463–141441259	14	33993	3	common	7(2)		MGAM	D7S2195
chr8:16256536–16692519	83	435984	1	rare	3(1)			
chr8:43592905–43910848	26	317944	3	common	4(1)			
chr9:71289871–71308782	10	18912	3	rare	15(11)			
chr10:44523027–44679489	27	156463	3	rare	15(8)			
chr10:47013328–47173619	35	160292	3	common	18(9)			
chr11:50343409–51228612	19	885204	3	rare	3(0)			
chr11:55127597–55204003	11	76407	1	common	19(9)	OR4C11,OR4C6,OR4P4,OR4S2		
chr12:7884583–8017012	26	132430	3	common	16(9)	SCL2A3	SLC2A14	
chr12:34466271–34711193	11	244923	3	rare	1(0)			
chr13:48903445–48961512	13	58068	1	rare^3^	5(2)		CAB39L,SETDB2	D11S153
chr13:61722125–62308067	107	585943	3	rare	22(7)			
chr13:68611259–68643623	13	32365	3	rare^3^	1(0)			
chr14:47841003–47920779	13	79777	1	rare^3^	4(0)			
chr14:91996705–92036873	20	40169	1	rare^3^	1(0)		SLC24A4	GATA31B
chr15:21905523–22023095	15	117573	1	common	10(7)			
chr15:32505886–32587887	11	82002	1	common	6(0)			
chr16:34326402–34550666	11	224265	3	common	8(2)			
chr16:69251501–69354016	27	102516	1	rare^3^	1(0)	ABBA-1	C16orf77,VAC14	
chr19:339413–371755	17	32343	1	common	1(1)		SHC2	
chr19:20422200–20520617	10	98418	1	common	11(4)			
chr20:61056151–61070635	11	14485	1	rare	2(1)		C20orf59	not reported
chr22:42613609–42627383	15	13775	1	rare^3^	1(0)		PNPLA5	

1 The chromosome coordinates are based on NCBI 36 human genome assembly.

2 The CN represent the different aberrant copy numbers observed in this pedigree (normal copy number is 2).

3 Marked are CNVs specific for Amish family 884.

4 Number of subjects (members of family 884) that have that CNV. In brackets are number of affected individuals.

5 Linkage markers that are within 2 Mbp of the CNVs, see [32] and references therein.

By comparing the CNVs detected in the Amish pedigree with those detected in a large control set of neurologically normal individuals [27] and with those in the database of genomic variants (http://projects.tcag.ca/variation), we identified 23 common CNVs present in the Amish family and in 1000 control subjects of European decent at a frequency of >1%. We found 27 CNVs present in <1% of subjects which were assigned as rare, 13 CNVs were found only in Amish family 884, and were therefore designated “Amish-specific” or “private” CNVs. (Table 1). The distribution of 50 CNVs in family members is shown in the heat map (Figure 1).

**Figure 2:**
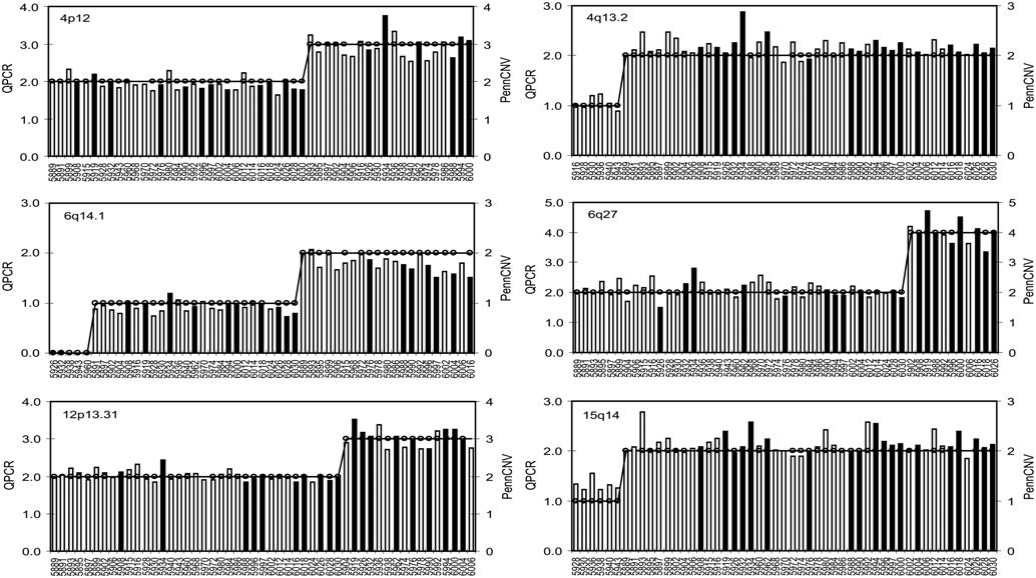
Validation of CNVs by QPCR in 51 individuals. The copy numbers detected by QPCR (bars) and PennCNV (circles) were plotted against all individuals in family 884 (only the last four digits of the cell line IDs were shown). Black bars or circles indicate affected individuals (including BPI, BPII, BP-NOS and MDD), white bars or circles indicate unaffected individuals. Note that the PennCNV calls were absent for subjects 5906, 5968, 5970, 6006, and 6024.

To experimentally validate the CNVs at a genomic level, we randomly selected 6 CNV regions (at 4p12, 4q13.2, 6q14.1, 6q27, 12p13.31, and 15q14; Table 1) and examined their copy number by real-time quantitative PCR (QPCR) in fibroblasts or LCLs from 51 Amish family members. The QPCR experiment confirmed the presence of these deletions or duplications in all subjects (Figure 2). Our results indicate that the CNVs detected by computational analysis of SNP genotyping data are highly reliable. Moreover, 19 CNVs were detected in family members across three generations and they were inherited in a Mendelian fashion with identical boundaries, suggesting that these variants are genetically stable.

### CNVs affect gene expression

To investigate a potential pathogenic role of these CNVs, we compared expression patterns of genes within and around these chromosomal rearrangements (with a 2 Mb sweep) in individuals with and without CNVs. Specifically, we examined the effects of 19 CNVs on gene expression using a combination of three methods: a) thirteen CNVs with different copy numbers in four individuals (GM05932, GM05934, GM05930 and GM05936) were examined using microarray expression data of fibroblasts and LCLs from these individuals; b) nine common CNVs (six of them were also included in the analysis of the four subjects described above) that also happened to exist in individuals from the Autism Genetic Resource Exchange (AGRE) collection, were analyzed using microarray expression data that are available in the public domain [27], [28]; c) four CNVs (including one examined in the microarray analysis of four subjects described above, which serves as a validation) were tested by QPCR in fibroblast samples from 48 individuals of Amish family 884 based on their higher frequency in affected subjects. These genotype-expression analyses revealed that, at a nominal P value <0.05, 58% of the tested CNVs (11 out of 19) were associated with expression changes of genes within, partially within or near these CNV regions in fibroblasts or LCLs (Supplemental Table S4 & S5).

Among 50 detected CNV regions, we focused on three regions due to their enrichment in affected subjects (≥70%), as well as their high frequency in family 884 (≥10 members) which will give reasonable power to detect genotype-expression association by QPCR: a common duplication on 6q27, a rare duplication on 9q21.11, and a common deletion on 15q11.2 (Table 1). Although a common duplication on 12p13.31 has a frequency of 56% in affected subjects, permutation test controlling for family structure revealed a trend toward significant association between CNV status and the disease status at this loci, along with a CNV on 15q11.2. (Supplemental Table S2). We found significant association between CNV status and expression of genes within or near these structural variants.

**Figure 3:**
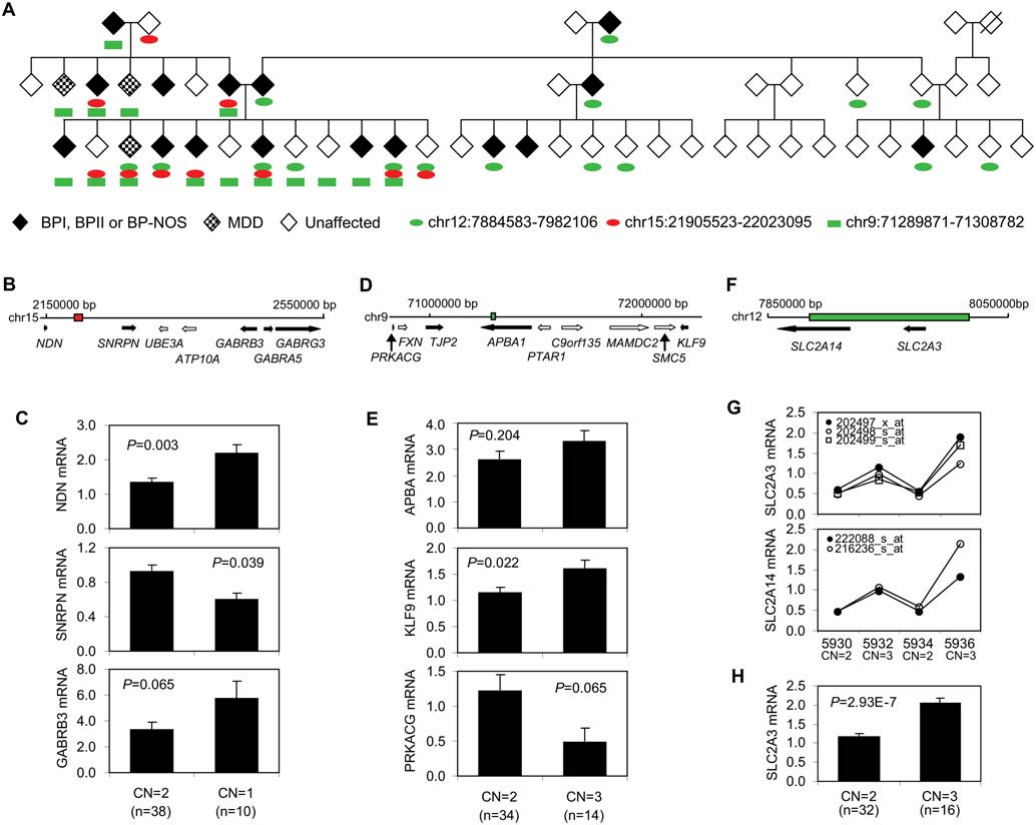
CNVs frequently found in affected subjects were associated with changes in gene expression. (A) The distribution of three CNVs in members of the Amish family 884. Ovals or bars below a subject indicate the presence of CNVs in that individual. Green indicates duplication by one copy, red indicates deletion by one copy. (B) Diagram of the locus of a CNV region (indicated by the red bar) in chr15 showing surrounding genes. Black arrows indicate genes examined in this study. (C) The CNV in chr15 was associated with altered expression of genes NDN (upper panel), *SNRPN* (middle panel), and *GABRB3* (lower panel). (D) Diagram of the locus of a CNV region (green bar) in chr9 located in the second intron of APBA1 gene. Black arrows indicate examined genes. (E) The CNV in chr9 does not affect the expression of *APBA1* gene (upper panel), but it was associated with altered expression of *KLF9* (middle panel) and *PRKACG* (lower panel). (F) Diagram of the locus of a CNV region (green bar) in chr12 containing gene *SLC2A3* and partially containing *SLC2A14*. (G) The expression levels of *SLC2A3* (top panels) and *SLC2A14* (lower panels) in LCLs of four individuals (GM05930, GM05932, GM05934, GM05936). Plotted are normalized data from microarray analysis for two or three probes. (H) The expression level of *SLC2A3* in 48 fibroblast samples from Amish family 884. CN, copy number. Data are mean±SE; *P* values were from t-test.

*Chr15: 21905523-22023095*: This common deletion (117.6 kb) was found in 10 subjects (seven affected including 6 BPD, and three unaffected; Figure 3A, B). Gene expression analysis using QPCR in 48 fibroblasts from Amish family 884 revealed that this CNV is associated with reduced expression of SNRPN (small nuclear ribonucleoprotein polypeptide N) located 596 kb telomeric of this CNV, and increased expression of *NDN* (necdin) located 422 kb centromeric of this CNV (*P* = 0.039 and 0.003, respectively; Figure 3B, C). The *SNRPN-SNURF* and *NDN* loci map to the Prader-Willi syndrome region and are imprinted - expressed from the paternal allele in brain tissues [29], [30]. We found that this deletion, when maternally inherited, is associated with an increase of *NDN* expression (*P* = 0.002, data not shown); however there were no significant changes in *NDN* or *SNRPN* when the deletion was paternally inherited (data not shown). Analysis of allele-specific transcription will be required to address the effect of this CNV on the expression and/or imprinting status of *NDN* and *SNRPN-SNURF* loci.

*Chr9: 71289871–71308782*: This duplication (18.9 kb) within the second intron of *APBA1* (amyloid beta A4 precursor protein-binding) was found in 15 individuals (four unaffected and eleven affected, including 8 BPD subjects; Figure 3A, D). Although we did not detect changes in expression of *APBA1* in fibroblasts (Figure 3E), this duplication was associated with an increase in expression of *KLF9* (Kruppel-like factor 9), located 910 kb telomeric of this CNV (*P* = 0.022, Figure 3E). *KLF9* is a transcription factor that binds to GC box elements of many promoters. This gene is expressed at a high level in the hippocampus, amygdala, and cerebellum. Moreover, disruption of this gene in the mouse results in a deficit in context-dependent fear conditioning [31].

*Chr6:168078929–168340091*: This common duplication (207 kb) was detected in 11 individuals (three unaffected, eight affected including 5 BPD) and encompasses *KIF25* (Kinesin family member 25, a member of the kinesin protein superfamily with a role in organelle transport and cell division) and *FRMD1* (FERM domain containing 1, function unknown), as well as the 3′ portion of the *MLLT4* (myeloid/lymphoid or mixed-lineage leukemia; translocated to, 4) gene (Supplemental Figure S2A, B). All three genes are expressed at low levels in fibroblasts and we were not able to detect changes in expression in individuals with this duplication (data not shown). We examined four genes (*SFT2D1*, *PRS6KA2*, *SMOC2*, *and THBS2*) that are located within a 2 Mbp region surrounding the boundaries of this CNV. The gene *SMOC2* (Secreted modular calcium-binding protein 2), located 244 kb telomeric of this CNV, has a higher expression in fibroblasts of individuals with the duplication (Supplemental Figure S2C).

*Chr12:7884583–8017012*: This common duplication significantly influenced gene expression in fibroblast cell lines. This chromosomal region (132.4 kb), duplicated in 16 individuals (seven unaffected, nine affected including 8 BPD), encompasses the entire *SLC2A3* gene and the first three exons of the paralogous *SLC2A14* gene (both genes encode neuronal glucose transporters) (Figure 3A, F). This duplication was passed from the grandfather (GM05962) to two affected and two unaffected children. Six out of ten affected and five out of 20 unaffected grandchildren have this rearrangement as well. Expression profiling in four Amish individuals (two with this CNV and two without) revealed that the *SLC2A3* and *SLC2A14* genes are expressed at a higher level in fibroblasts and LCLs of individuals with an extra copy of this region (Figure 3G and Supplemental Table S4). The CNV's effect on the *SLC2A3* expression was further validated by QPCR in 48 fibroblasts from Amish family 884 (*P* = 2.93E-7, t-test, Figure 3H).

The genotype-expression analysis supports previous reports that a substantial portion of CNVs may affect gene expression. We show that selected CNVs, frequently observed in affected members of the 884 family, correlate with changes in the level of expression of specific genes, located within or around the rearrangement. However, despite their role in regulating expression of neuronal genes and their enrichment in family members with affective disorders, our work does not establish that any of the observed CNV is the sole cause of bipolar disease in this pedigree, although their potential role as risk factors can not be ruled out. Further work is warrant to confirm or refine these findings in additional family members and unrelated individuals.

## Discussion

Structural variation including CNV constitutes a substantial portion of total genetic variability and is important in understanding the biology of common disease. Using high-density arrays we identified 50 deletions and duplications (with a threshold of >10 SNPs) in the large Amish pedigree segregating BPD and related mental disturbances. Although, the average number and size of detected deletions and duplications in each individual did not differ significantly between affected and unaffected family members nor between this family and other ethnic groups of European decent [27]. We show that 58% of the CNVs assayed in our report are associated with changes in gene expression and can be detected in one or both of two peripheral cell lines (fibroblasts and LCL). Our analysis illustrates an advantage of using a combination of cell lines to estimate pathogenic effects of chromosomal rearrangements.

Extensive genetic studies in BPD have implicated a number of susceptibility loci (reviewed in [32]); several collaborative efforts have recently published whole-genome association studies with BPD [33]–[36]. Although our analysis did not identify CNVs (with a size ≥10 SNP threshold) in regions of these modest association peaks or linkage peaks on chromosome 16, 13 and 15 that were previously reported in this pedigree [22], several CNVs detected in our study map in the vicinity of linkage peaks in other BPD studies (Table 1). Functional studies of these variants will be needed to evaluate their role in disease susceptibility. Several common CNVs have been reported to be associated with complex diseases (reviewed in [37]), however, these CNVs were identified by candidate gene approaches instead of genome-wide and hypothesis-free surveys [37]. Recent genome-wide studies in autism, schizophrenia and Amyotrophic lateral sclerosis (ALS) suggest that multiple rare deletions and duplications should be considered as potential disease-predisposing factors [9], [38], [39]. Given the complex nature of BPD and the substantial contribution of CNVs to genetic heterogeneity, genome-wide survey of genomic aberrations including common and low frequency (or rare) variants may compensate traditional genetic analysis of BPD. A candidate gene-driven analysis revealed a higher frequency of a CNV in BPD, which was predicted to disrupt *GSK3B* gene [40]. The only reported genome-wide copy number analysis in BPD has been done using BAC array comparative genome hybridization (aCGH) at 1.4 Mbp resolution; three CNVs containing genes involved in glutamate signaling were identified in 5 or less out of 50 BPD subjects [41]. However, the significant size-overestimation of CNVs identified by BAC aCGH [42] indicate that a more systematic approach is needed to detect CNVs in BPD. Although small in scale and limited to one large pedigree, our analysis of CNVs in the Old Order Amish pedigree represents the first high resolution genome-wide survey of CNVs in BPD.

A unique aspect of our study is the family-based multigenerational analysis, which permits examination of the inheritance patterns of the CNVs and facilitates validation of computationally predicted CNVs. More importantly, our study provides a proof of principle for a family-based investigation of a summation or combination of a series of common and rare CNVs involving different genes and genomic regions that could confer low or moderate risk for a disease. For example, a larger pedigree would afford more power to identify individuals sharing not only the same rare structural variant, but in some cases, the same combination of variants involving different genes or intergenic regions. Furthermore, as implicated by our analysis of CNV's effect on *SNRPN* and *NDN* expression, epigenetic modifications and an imbalance in the level of expression between two homologous chromosomes may lead to marked differences in the effect of a deletion or duplication. Measurements of an allelic imbalance in multiple family members carrying the same rare structural variant, on either the maternal or paternal chromosome, provide a unique advantage of family-based genotype-expression-phenotype analysis.

Our study includes extensive experimental validation of detected CNVs, as well as analysis of the effect of a subset of randomly selected CNVs on gene expression. Although a recent study of gene expression in LCLs of four ethnic groups (each consisting of 45–60 individuals) revealed a substantial effect of CNVs on gene expression and predicted that CNVs account for at least 17.7% of genetic variation in gene expression [7], we evaluate effects of specific CNVs, some of them frequently found in affected family members. Previous analyses of CNVs in human diseases rarely investigated the potential contribution of the CNVs through regulating expression of surrounding genes, which account more than half of the CNV regulated genes in LCLs [7]. Our genotype-expression analysis of 19 CNVs in two cell lines (LCLs and fibroblasts) showed that the majority of changes were detected in genes outside the deleted or duplicated regions (for example, *SNRPN*, *NDN* and *KLF9*). These findings illustrate that for the evaluation of the functional role of CNVs it will be critical to have deeper insight into regulatory elements in the intergenic and intronic regions affected by these rearrangements. Although caution is required to conclude any causal role of the CNV-induced gene expression change in the etiology of BPD, they may serve as candidate potential risk factors warrant further study.

The limitation of our study is the relatively small subject size, which resulted in no significant association between CNVs and BPD. Future studies should include the entire Old Order Amish family 884 (over 400 individuals), along with the fourth generation which is already enroled in this longitudinal and prodromal study [43]. Furthermore, analysis of low frequency variants in additional large families and different ethnic groups may increase the pool of variants. Another limitation of our study is the use of cell lines, rather than brain tissues, for expression analysis. Although we found that 58–71% of 2563 neuronal genes (3077 unique transcripts represented by 3185 probes on the expression array) are expressed in two peripheral cell lines (LCLs and fibroblasts respectively, Supplemental Figure S3), the effect of CNVs on gene expression may differ in these cell lines in comparison to brain tissues. It is therefore critical to extend the genotype-expression analysis to neuronal tissues, such as sections of postmortem brain regions or olfactory epidermal biopsies, to investigate the functional role of these genetic traits in the pathogenesis of affective disorders.

In summary, we identified 50 CNV regions in the Amish family 884 segregating affective illness, particularly BPD. Functional annotation of the genes affected by common, rare and “Amish-specific” or “private” CNVs will provide an important avenue towards establishing molecular links to complex clinical phenotypes, such as affective disorders in this family. Our results support the concept that BPD, even in a genetically isolated family (Amish family 884), is a complex disorder and a combination of multiple genetic lesions likely contribute to its pathogenesis. Our data also suggest that studying the functional consequence of CNVs by examining their effects on gene expression may shed new lights to dissecting the etiology of affective disorders including BPD.

## Materials and Methods

### Cell culture and isolation of DNA and RNA

Forty-eight fibroblast cell lines and seven lymphoblastoid cell lines (LCLs) (Supplemental Table S1) from the Old Order Amish family 884 were purchased from Coriell cell repositories (Coriell Institute for Medical Research, Camden, NJ). The cells were cultured according to the provider's recommendation as previously described [44]. The genomic DNA was isolated using a Genomic DNA Purification Kit (Gentra Systems, Minneapolis, Minnesota). Total RNA was isolated by Trizol reagent as described previously [45], from which the cDNA was generated using the high-capacity cDNA Reverse Transcription Kit (Applied Biosystems, Foster City, CA).

### Genotyping and identification of copy number variant (CNV)

The genomic DNA from fibroblasts or LCLs was used to obtain genotypes by the Illumina HumanHap550 version 3 high-density arrays with 561,446 SNP markers. The genotyping experiments were performed at the Center for Applied Genomics, Children's Hospital of Philadelphia as previously described [46]. The raw genotyping signal data were processed by the Illumina BeadStudio software and converted to signal intensity values, represented as Log R Ratio (LRR) and B Allele Frequency (BAF). Due to the presence of “genomic wave patterns” in some of the genotyped samples, we applied a data pre-processing protocol [47] to increase the signal-to-noise ratio of the LRR values for all samples. We have confirmed in three individuals that the SNP genotypes using DNA from fibroblasts were nearly identical to those obtained using DNA from LCLs of the same individual (concordance rate >99.99%), thus justifying the combined analysis of genotyping data from these two types of cells.

A previously described high-resolution CNV detection algorithm, the PennCNV algorithm [25], was used to infer CNVs from the signal intensity data. This algorithm incorporates multiple sources of information, including total signal intensity and allelic intensity ratio at each SNP marker, the distance between neighboring SNPs, the allele frequency of SNPs, as well as family information when available. By taking advantage of the pedigree structure, we split the large family into quartets and trios and applied the posterior validation procedure in PennCNV algorithm for more accurate CNV detection and boundary mapping. We set a threshold at 10 SNPs to avoid false positive calls. This threshold was previously shown to result in a false positive rate lower than 1% for high-quality samples [25], [26].

To define common and rare CNVs we mapped these CNVs to the UCSC genome browser for comparison with those previously identified in other publications [9], [27], or with those included in the database of genomic variants (http://projects.tcag.ca/variation). The common and rare CNVs were defined as those that occurred at a frequency of >1% or <1% in general populations. The CNVs which were not detected in control subjects or in the database of genomic variants were considered “Amish-specific” or “private”.

To identify CNVs that are prevalent in affected and/or unaffected subjects, we performed permutation test for each CNV. (see Supporting Text S1)

### Microarray and CNV-expression association

Five µg of total RNA from fibroblasts and LCLs of four individuals (GM05931, GM05933, GM05935, and GM05937) were subjected to microarray experiments using the HG U133A 2.0 Array (Affymetrix, Santa Clara, CA). These four individuals are siblings within a large sibship; two of them have BPD (BPI). Data processing was carried out as described previously [45]. Briefly, Affymetrix Microarray Suite 5.0 was used to quantitate expression levels and assign Calls (Flags of present, marginal, and absent) for each probe set. The CEL files were used to generate normalized expression data that were corrected by the GCRMA algorithm. The normalized expression data for probes located within a 2 Mb region surrounding each boundary SNP of a CNV were subjected to association study. A gene was considered to be regulated by a CNV if its expression levels in fibroblasts or LCLs correlated (or inversely correlated) with the copy numbers of respective CNVs (regression *P*<0.05) and there was >1.5 fold change in their expression levels among the four individuals. The microarray expression data have been deposited to GEO (GSE11767).

In addition, we retrieved from GEO the expression profiling data for 30 LCLs (GSE7329) which were included in a study for autism [28]. We have also genotyped these 30 LCLs by the same Illumina HumanHap550 platform with the same genotyping protocol for the Amish families [27]. From these 30 LCLs, we identified nine CNVs using the PennCNV algorithm that also exist in Amish family 884 and were present in at least three of these 30 individuals. Regression analysis was performed on expression data for genes located within a 2 Mb region flanking the boundaries of these eight CNVs, and the genes yielding a *P*<0.05 were considered to be potentially associated with the corresponding CNV.

### CNV validation and gene expression quantification

We employed real-time quantitative PCR (QPCR) using relative quantification method with SYBR Green Dye to validate CNVs and to quantify gene expression. Six CNVs were randomly selected and PrimerExpress2.0 software was used to design primer pairs that target the CNV regions. The endogenous control for CNV validation was designed to target a region in chromosome 12 within the DEC2 gene that is free of any known structural variants in both Amish family 884 and in the general population. The primer pairs for gene expression were designed to target exon-exon junctions if the target gene contains multiple exons. The endogenous control for gene expression analysis was β-actin. The sequences for these primers are listed in Supplemental Table S6.

A different batch of genomic DNA was isolated from fibroblasts or LCLs of all the 51 individuals in Amish family 884 and subjected to CNV validation. For gene expression quantification, cDNA samples prepared from 48 fibroblast cell lines were examined. The real-time PCR was done on the ABI Prism 7900HT system (Applied Biosystems, Foster City, CA) and the data were analyzed as described previously [44]. The relative quantity of the genomic copy numbers or the gene expression levels was normalized to GM05889, an individual that has none of the CNVs examined, and was set as a copy number of two for all CNV regions and a gene expression level of 1 for all transcripts.

## Supporting Information

Text S1 Permutation Tests for Association Between CNV status and Phenotype
(0.09 MB DOC)

Table S1 Cell lines and Disorder versus Normal groups for the phenotype-genotype association analysis
(0.16 MB DOC)

Table S2 P-values of odds ratios for individual CNV regions
(0.25 MB DOC)

Table S3 P-values of adjusted ratios
(0.08 MB DOC)

Table S4 Genes with expression levels associated with CNVs (indicated by chromosomal coordinates in bold) in fibroblasts and LCLs of 4 Amish individuals
(0.08 MB DOC)

Table S5 Genes with expression levels associated with CNVs (indicated by chromosomal coordinates in bold) in LCLs of 30 AGRE individuals (Nishimura, Y., et al. 2007 Hum Mol Genet 16, 1682–98)
(0.04 MB DOC)

Table S6 Primer sequences corresponding to genomic or cDNA regions for CNV validation and gene expression by QPCR
(0.05 MB DOC)

Figure S1 Distribution of 32 CNVs present in four or more individuals in family 884. Colored ovals indicate the presence of CNV in that individual. The number of copies are indicated by different colors.
(5.18 MB EPS)

Figure S2 A duplication in chromosome 6 correlates with changes in SMOC2 gene expression. (A) The distribution of this duplication (two copies) in Family 884. Marked are subjects with this CNV. (B) Diagram of the locus of this CNV region in chr6. Genes examined in this study are indicated by black arrows. (C) This CNV is associated with increased expression of SMOC2, but not MLLT4, in fibroblasts. The expression of genes KIF25 and FRMD1 were not detectable.
(1.02 MB EPS)

Figure S3 Fibroblasts and LCLs express a large number of neuronal genes. (A) Venn diagram showing the number of genes expressed in fibroblasts and LCLs (genes present or marginal in all four individuals by expression profiling using the Affymetrix HG_U133A_2 arrays), and the overlap between neuronal genes (genes expressed >4 fold higher in any of the 23 neuronal tissues than the 52 non-fetal non-neuronal tissues in GNF data [Su AI, Cooke MP, Ching KA, Hakak Y, Walker JR, et al. (2002) Large-scale analysis of the human and mouse transcriptomes. Proc Natl Acad Sci U S A 99: 4465–4470. ]). Expression of 71.0% neuronal genes can be detected in fibroblasts, 58.0% in LCLs, and 24.6% neuronal genes were not detectable in any of these cell lines (B) The hierarchical clustering of neuronal gene expression in fibroblasts (left) and LCLs (right) in four individuals using GeneSpring 7.2 software. The color bar indicates normalized expression level. More neuronal genes are expressed at high levels in fibroblasts than in LCLs, indicating that fibroblasts maybe better suited than LCLs for studying neurological disease. However, most of the neuronal genes tend to be highly expressed in either fibroblasts or LCLs, but not both, suggesting that fibroblasts and LCLs can complement each other in studying neuronal gene expression.
(1.32 MB EPS)
