# Supplementary material for: Genomic Landscape of a Three-Generation Pedigree Segregating Affective Disorder
Source: PLoS One. 2009 Feb 13;4(2):e4474. doi: 10.1371/journal.pone.0004474 (PMC2637422; doi:10.1371/journal.pone.0004474)
Supplement: Text S1 — Permutation Tests for Association Between CNV status and Phenotype (0.09 MB DOC) [file pone.0004474.s001.doc]

# Text S1. Permutation Tests for Association Between CNV status and Phenotype

### Definitions and Procedures

The large pedigree size precludes the use of standard methods for the family-based association test. Therfore, we performed a permutation procedure that adjusts the sibship relationships, at the potential cost of decreased power. We used 40 non-founder subjects with good-quality genotype data in our dataset for the permutation analysis. Given any test statistic S estimating the association between phenotype and genotype, its p-value is determined by the following permutation procedure. To shuffle the phenotype labels, we divide the individuals into groups that share the same parents (there are no half-siblings in this dataset), and shuffle the phenotype labels within each group to obtain the new phenotype assignment. We permute 10000 times and compute the corresponding test statistic values, S_1_ through S_10000_. The two-sided p-value of the test statistic (always a ratio in our scenario) based on the actual phenotype assignment, S_0_, is the proportion of the 10000 randomized statistics that are more extreme:

If S0>1, then P(S_0_) = (Number of S_i_’s ≥S_0_ + Number of S_i_’s ≤1/S_0_, 1≤i≤10000)/10000

If S0<1, then P(S_0_) = (Number of S_i_’s ≥1/S_0_ + Number of S_i_’s ≤S_0_, 1≤i≤10000)/10000

We analyzed six groupings; see the table following this text.

We evaluated the following statistics:

1. Odds ratio of the 2-by-2 contingency matrix for genotype (has CNV vs. no CNV) and phenotype (Normal vs Disorder). For each locus with copy-number variations, we only include subjects of whom at least one parent has copy-number variation at the locus, i.e., the subjects’ CNVs are inherited. The odds ratio is computed by the function fisher.test() from the R software using the 2-by-2 contingency table (phenotype versus CNV status). Only CNV regions where at least five subjects with CNVs and at least five subjects without CNVs in are tested.
2. Adjusted ratios. Let I_N_ and I_D_ be the normal and disorder subsets of the 40 subjects we examined; let N_N_ and N_D_ be the number of subjects in I_N_ and I_D_, respectively. Let K_5_ be the set of CNV regions such that for each of these CNV regions, the number among the 40 subjects having CNVs in the region is at least 5 (there are 15 such CNVs). Let K_5,dup_ (K_5,del_) be the subset of K_5_ with CNVs that have duplications (deletions) only; this is well-defined because CNVs are either duplication-only or deletion-only in this dataset. Let *C_ij_* be the number of copies of CNV region *j* in individual *i* (can be 0 (double deletion), 1 (single deletion), 2 (normal), 3 (single duplication), or 4 (double duplication)). Given any particular weighting scheme *w*, we tested the following three adjusted ratios:

,

,

.

- 1. (**Cnv**) adjusted ratio of the number of CNVs: *w_j_*=1 for all CNV region *j*.
  2. (**Size**) adjusted ratio of the total length of CNVs: for any CNV region *j*, *w_j_* is its length by the number of nucleotides.
  3. (**Gene cnt**) adjusted ratio of the number of genes intersected with CNV regions: for any CNV region *j*, *w_j_* is the number of Refseq genes intersecting with the region.
