# Supplementary material for: Genomic Landscape of a Three-Generation Pedigree Segregating Affective Disorder
Source: PLoS One. 2009 Feb 13;4(2):e4474. doi: 10.1371/journal.pone.0004474 (PMC2637422; doi:10.1371/journal.pone.0004474)
Supplement: Table S1 — Cell lines and Disorder versus Normal groups for the phenotype-genotype association analysis (0.16 MB DOC) [file pone.0004474.s002.doc]

### Table S1. Cell lines and Disorder versus Normal groups for the phenotype-genotype association analysis

| **Fibroblast**^1^ | **LCL**^1^ | **Is Founder** | **Bad Genotype Quality** | **CNV#**^2^ | **Age**^3^ | **Diagnosis**^4^ | **Analysis Grouping^5^** | | | | | |
| --- | --- | --- | --- | --- | --- | --- | --- | --- | --- | --- | --- | --- |
|  |  |  |  |  |  |  | **1** | **2** | **3** | **4** | **5** | **6** |
| GM05908 |  |  |  | 13 | 24 | BPI | D | D | D | D | D | D |
| GM05926 |  |  |  | 10 | 50 | BPI | D | D | D | D | D | D |
| GM05932 |  |  |  | 6 | 25 | BPI | D | D | D | D | D | D |
| GM05934 |  |  |  | 9 | 23 | BPI | D | D | D | D | D | D |
| GM05976 |  |  |  | 12 | 26 | BPI | D | D | D | D | D | D |
| GM05988 |  |  |  | 6 | 30 | BPI | D | D | D | D | D | D |
| GM05990 |  |  |  | 5 | 25 | BPI | D | D | D | D | D | D |
|  | GM05997 |  |  | 6 | 18 | BPI | D | D | D | D | D | D |
| GM06000 |  |  |  | 9 | 22 | BPI | D | D | D | D | D | D |
| GM06004 |  |  |  | 8 | 16 | BPI | D | D | D | D | D | D |
| GM06016 |  | Y |  | 6 | 74 | BPI |  |  |  |  |  |  |
| GM06028 |  |  |  | 8 | 41 | BPI | D | D | D | D | D | D |
| GM06030 |  |  |  | 5 | 37 | BPI | D | D | D | D | D | D |
| GM05962 |  | Y |  | 9 | 86 | BPII |  |  |  |  |  |  |
| GM05996 |  |  |  | 7 | 50 | BPII | D | D | D | D | D | D |
| GM05994 |  |  |  | 7 | 52 | BP-NOS, Cyclothymic | D | D | D | D | D | D |
| GM05919 |  |  |  | 11 | 26 | MDD | D | D | D |  |  |  |
| GM06018 |  |  |  | 8 | 39 | MDD, situational | D | D | D |  |  |  |
| GM06026 |  |  |  | 5 | 46 | MDD, situational | D | D | D |  |  |  |
| GM05970 |  |  | Y |  | 61 | MiDD |  |  |  |  |  |  |
| GM05972 |  |  |  | 9 | 58 | MiDD | N |  |  | N |  |  |
| GM06006 |  |  | Y |  | 12 | MiDD |  |  |  |  |  |  |
| GM06024 |  |  | Y |  | 47 | MiDD |  |  |  |  |  |  |
| GM05902 |  |  |  | 8 | 28 | MiDD symptom | N |  |  | N |  |  |
| GM05992 |  |  |  | 7 | 20 | postpartum psychosis | N | N |  | N | N |  |
| GM05960 |  | Y |  | 20 | 82 | Labile Personality, probable |  |  |  |  |  |  |
| GM05895 |  |  |  | 10 | 22 | N early signs of BP | N | N | N | N | N | N |
| GM05889 |  |  |  | 4 | 18 | N | N | N | N | N | N | N |
| GM05891 |  |  |  | 11 | 16 | N | N | N | N | N | N | N |
| GM05893 |  |  |  | 7 | 20 | N | N | N | N | N | N | N |
| GM05897 |  |  |  | 8 | 44 | N | N | N | N | N | N | N |
| GM05899 |  | Y |  | 6 | 45 | N |  |  |  |  |  |  |
| GM05904 |  |  |  | 6 | 56 | N | N | N | N | N | N | N |
| GM05906 |  |  | Y |  | 33 | N |  |  |  |  |  |  |
| GM05915 |  |  |  | 7 | 23 | N | N | N | N | N | N | N |
|  | GM05916 |  |  | 9 | 20 | N | N | N | N | N | N | N |
| GM05928 |  | Y |  | 9 | 48 | N |  |  |  |  |  |  |
| GM05930 |  |  |  | 11 | 26 | N | N | N | N | N | N | N |
| GM05936 |  |  |  | 13 | 18 | N | N | N | N | N | N | N |
| GM05938 |  |  |  | 11 | 16 | N | N | N | N | N | N | N |
| GM05940 |  |  |  | 12 | 14 | N | N | N | N | N | N | N |
|  | GM05943 |  |  | 10 | 12 | N | N | N | N | N | N | N |
| GM05968 |  | Y | Y |  | 82 | N |  |  |  |  |  |  |
| GM05974 |  |  |  | 7 | 55 | N | N | N | N | N | N | N |
| GM05978 |  |  |  | 12 | 17 | N | N | N | N | N | N | N |
| GM05980 |  |  |  | 6 | 15 | N | N | N | N | N | N | N |
| GM05984 |  |  |  | 7 | 27 | N | N | N | N | N | N | N |
| GM05986 |  |  |  | 8 | 20 | N | N | N | N | N | N | N |
| GM06002 |  |  |  | 10 | 19 | N | N | N | N | N | N | N |
| GM06012 |  |  |  | 5 | 33 | N | N | N | N | N | N | N |
| GM06014 |  | Y |  | 5 | 77 | N |  |  |  |  |  |  |

^1^ Cell lines derived from these subjects were purchased from Coriell Institute for Medical Research (Camden, NJ).

^2^ The number of CNVs (>=10SNP) detected in each cell line. Blank indicates information not available due to low quality of DNA or genotype.

^3^ The age of the subjects when the blood or biopsies were taken for cell line establishment.

^4^ The clinical diagnosis of the subjects. N, clinically normal; BPI, bipolar type I; BPII, bipolar type II; BP-NOS, bipolar disorder – not otherwise specified; MDD, major depression; MiDD, minor depression.

^5^ Affected (D) and unaffected (N) subjects included in the permutation analysis.
