# Supplementary material for: Genomic Landscape of a Three-Generation Pedigree Segregating Affective Disorder
Source: PLoS One. 2009 Feb 13;4(2):e4474. doi: 10.1371/journal.pone.0004474 (PMC2637422; doi:10.1371/journal.pone.0004474)
Supplement: Table S2 — P-values of odds ratios for individual CNV regions (0.25 MB DOC) [file pone.0004474.s003.doc]

**Table S2.** P-values of odds ratios for individual CNV regions.

Only CNV regions where at least five subjects with CNVs and at least five subjects without CNVs in are tested; otherwise the entries are grayed out.
