# Supplementary material for: Genomic Landscape of a Three-Generation Pedigree Segregating Affective Disorder
Source: PLoS One. 2009 Feb 13;4(2):e4474. doi: 10.1371/journal.pone.0004474 (PMC2637422; doi:10.1371/journal.pone.0004474)
Supplement: Table S3 — P-values of adjusted ratios (0.08 MB DOC) [file pone.0004474.s004.doc]

### Table S3. P-values of adjusted ratios
