# Supplementary material for: Genomic Landscape of a Three-Generation Pedigree Segregating Affective Disorder
Source: PLoS One. 2009 Feb 13;4(2):e4474. doi: 10.1371/journal.pone.0004474 (PMC2637422; doi:10.1371/journal.pone.0004474)
Supplement: Table S4 — Genes with expression levels associated with CNVs (indicated by chromosomal coordinates in bold) in fibroblasts and LCLs of 4 Amish individuals (0.08 MB DOC) [file pone.0004474.s005.doc]

**Table S4.** Genes with expression levels associated with CNVs (indicated by chromosomal coordinates in bold) in fibroblasts and LCLs of 4 Amish individuals.

^*^ Regression *P* values, blank indicates the absence of expression in all 4 individuals. In bold are significant values (*P*<0.05) and in *Italic* are values keep significant after Bonferroni adjustment.

| Gene Symbol | Probe ID | *P*-fibroblast^*^ | *P*-LCL^*^ |
| --- | --- | --- | --- |
| **chr2:41083802-41099005 (0,1,1,0)^†^** | | |  |
| EML4 | 220386_s_at | 0.96282 | **0.01543** |
| **chr2:89714801-89877778 (1,2,1,1)** | | |  |
| PLGLA1 | 205871_at | **0.01272** | 0.25103 |
| RPIA | 212973_at | **0.04279** | 0.47543 |
| IGKC | 214669_x_at |  | 0.60322 |
| IGKC | 214768_x_at |  | ***0.00009*** |
| IGKC | 211643_x_at |  | ***0.00706*** |
| **chr6:79029920-79088461 (1,0,1,1)** | | |  |
| ELOVL4 | 219532_at | ***0.00422*** | ***0.00044*** |
| **chr7:100715342-100914175 (2,2,2,3)** | | |  |
| ZNF394 | 214714_at | 0.81887 | **0.00471** |
| CYP3A4 | 205998_x_at | 0.82970 | 0.42037 |
| CYP3A4 | 208367_x_at |  | **0.04881** |
| CYP3A4 | 205999_x_at |  | **0.01012** |
| MCM7 | 208795_s_at | 0.04499 | 0.45984 |
| MCM7 | 210983_s_at | 0.11500 | 0.72347 |
| PVRIG | 219812_at |  | **0.02601** |
| PILRA | 219788_at |  | ***0.00011*** |
| PILRA | 222218_s_at |  | 0.09088 |
| SERPINE1 | 202627_s_at | **0.02647** |  |
| AP1S1 | 209635_at | 0.37104 | 0.05914 |
| AP1S1 | 205195_at | 0.93694 | **0.04208** |
| AP1S1 | 205196_s_at | 0.43433 | 0.08223 |
| PLOD3 | 202185_at | **0.04550** | 0.30427 |
| PRKRIP1 | 218378_s_at | 0.68239 | **0.01308** |
| ORAI2 | 218812_s_at | **0.00521** | 0.05316 |
| **chr11:55127597-55204003 (1,1,1,2)** | | |  |
| SLC43A3 | 213113_s_at | 0.52101 | **0.04614** |
| SLC43A3 | 210692_s_at | 0.05306 | 0.18746 |
| UBE2L6 | 201649_at | 0.82678 | **0.01787** |
| **chr12:7884583-8017012 (2,3,2,3)** | | |  |
| TAPBPL | 218746_at | 0.05855 | 0.11894 |
| TAPBPL | 218747_s_at | **0.00643** | **0.01609** |
| VAMP1 | 213326_at | 0.17211 | 0.42386 |
| VAMP1 | 207100_s_at | 0.38772 | 0.33846 |
| VAMP1 | 207101_at | 0.97166 | **0.01711** |
| SLC2A14 | 222088_s_at | 0.22190 | 0.05798 |
| SLC2A14 | 216236_s_at | 0.56981 | 0.18784 |
| SLC2A3 | 202497_x_at | 0.45472 | 0.12810 |
| SLC2A3 | 202498_s_at | 0.18877 | **0.03764** |
| SLC2A3 | 202499_s_at | 0.40668 | 0.22098 |
| **chr15:32505886-32587887 (1,2,2,1)** | | |  |
| SCG5 | 203889_at | 0.77883 | **0.00456** |
| GOLGA8B | 208797_s_at | **0.03020** | 0.32310 |
| GOLGA8B | 210425_x_at | 0.17751 | 0.30798 |
| GOLGA8A | 208798_x_at | **0.04717** | 0.84850 |
| GOLGA8A | 210424_s_at | 0.13006 | 0.44309 |
| GOLGA8A | 213650_at | 0.63381 | 0.23135 |
| **chr4:47424121-47631300 (3,2,3,3)** | | |  |
| **chr4:66521854-66632070 (1,2,2,1)** | | |  |
| **chr4:161258794-161290832 (1,2,1,1)** | | |  |
| **chr6:67075448-67105019 (1,2,1,1)** | | |  |
| **chr6:124467336-124510591 (3,3,2,3)** | | |  |
| **chr16:34326402-34550666 (2,2,3,3)** | | |  |

^†^ The chromosome coordinates of CNVs (NCBI 36 human genome assembly) are followed by their copy numbers (in parenthesis) in 4 individuals (GM05930, GM05932, GM05934, GM05936).
