# Supplementary material for: Genomic Landscape of a Three-Generation Pedigree Segregating Affective Disorder
Source: PLoS One. 2009 Feb 13;4(2):e4474. doi: 10.1371/journal.pone.0004474 (PMC2637422; doi:10.1371/journal.pone.0004474)
Supplement: Table S5 — Genes with expression levels associated with CNVs (indicated by chromosomal coordinates in bold) in LCLs of 30 AGRE individuals (Nishimura, Y., et al. 2007 Hum Mol Genet 16, 1682–98) (0.04 MB DOC) [file pone.0004474.s006.doc]

**Table S5.** Genes with expression levels associated with CNVs (indicated by chromosomal coordinates in bold) in LCLs of 30 AGRE individuals (Nishimura, Y., *et al*. 2007 Hum Mol Genet 16, 1682-98).

| Gene Symbol | Probe ID | *P*-value^*^ |
| --- | --- | --- |
| **chr2:41083802-41099005^†^** | | |
| MAP4K3 | 42003 | 0.0516 |
| MAP4K3 | 16219 | **0.0084** |
| TMEM178 | 5017 | **0.0231** |
| SLC8A1 | 28985 | **0.0188** |
| SLC8A1 | 4135 | 0.8594 |
| SLC8A1 | 20183 | **0.0341** |
| SLC8A1 | 12999 | **0.0074** |
| COX7A2L | 40535 | 0.1067 |
| COX7A2L | 31059 | **0.0199** |
| **chr7:141407267-141441259** | | |
| C7orf34 | 7896 | 0.3817 |
| C7orf34 | 5755 | **0.0190** |
| ZYX | 8280 | **0.0216** |
| **chr11:55127597-55204003** | | |
| OR5AK2 | 32198 | **0.0283** |
| PRG2 | 32075 | **0.0346** |
| **chr15:32505886-32587887** | | |
| C15orf45 | 26108 | **0.0324** |
| GOLGA8A | 42780 | 0.0910 |
| GOLGA8A | 37364 | 0.1681 |
| GOLGA8B | 10075 | **0.0031** |
| **chr8:43592905-43910848** | | |
| **chr6:67075448-67105019** | | |
| **chr6:79029920-79088461** | | |
| **chr16:34326402-34550666** | | |

^*^ Regression *P* values. Bolded are significant (*P*<0.05).

^†^ Coordinates of the CNV regions based on NCBI 36 human genome assembly.
