# Supplementary material for: Genomic Landscape of a Three-Generation Pedigree Segregating Affective Disorder
Source: PLoS One. 2009 Feb 13;4(2):e4474. doi: 10.1371/journal.pone.0004474 (PMC2637422; doi:10.1371/journal.pone.0004474)
Supplement: Table S6 — Primer sequences corresponding to genomic or cDNA regions for CNV validation and gene expression by QPCR (0.05 MB DOC) [file pone.0004474.s007.doc]

**Table S6**. Primer sequences corresponding to genomic or cDNA regions for CNV validation and gene expression by QPCR.

| Gene or CNV | Forward primer | Reverse primer |
| --- | --- | --- |
| Cont-chr12-26M | CCCACCCCCATCCCTTC | TGGGCGATTTCAGAGATCG |
| chr4-66.5M | AGCAAATTAGCCCCTGTCTCC | TGCTTTTATTATGTAGTCCTAGGCCAA |
| chr6-79M | CCTGAGGCTTTCTTTCTCATTTCT | CCTCAAACACTCTATATCTTTATTTGCAGA |
| chr15-32.5M | CCATCAGCCATCTCCACCAT | TGAAATTCCTGAAAAGCAAGCC |
| chr6-168M | TTTCCCGCAGGCTCATTTT | ACTTCAACCTTTGGGCTTCTTG |
| chr12-7.9M | GTTACAAAACGTGACTCGGTTCAT | GCTTGTCTTGCTTACGGAGGG |
| chr4-47M | TTATTTAGGCTGAACTAGAAGCTTTTGA | TCCGACGACCCTTCAGTCTG |
| ACTB (control) | AGGCACCAGGGCGTGAT | GCCCACATAGGAATCCTTCTGAC |
| SLC2A3 | CCCTCCGCTGCTCACTATTT | GCCGGTGAAGATAATAAAAACGTAG |
| APBA1 | AACCTACGTTGAAGTTCCGGG | CCATCGATCAAGTCTTCGGG |
| KLF9 | CCCATCTCAAAGCCCATTACA | AGGGAAAGGGCCGTTCAC |
| TJP2 | ACGCCCCAGCACACGA | TGGAAGGAGCTTTCTGTGGCT |
| FRMD1 | TCGAGCCACACTCCTACTTCC | CAATCCCCCTCTTGGTGATG |
| KIF25 | GCTCTTCAGGCTCATTTTGGA | ACTTCAACCTTTGGGCTTCTTG |
| MLLT4 | AGCGTGTTACACGTTCCCAAG | CAAGTTGGTAAGCTTTATCTTCTCGAA |
| SFT2D1 | TGTGTGCTGCTCTTTGGTGG | TGCAGAATAACACAGCCAGTCC |
| RPS6KA2 | AGGACGTGCACCTGGTGAAG | TTAGAGCAAAGTAGGTGGCGG |
| SMOC2 | ACCTGTACAAGGGCCGCC | CATGCTTTTTGGCACCCG |
| THBS2 | GGACGATGTCTTCAATGAACGA | TGGTCAGTGTTGTAGACGTAGGGA |
| FSTL5 | TTTTTCATTCCCACCACAACACT | AAATCCAAACCTCATATGGGTGA |
| RAPGEF2 | CATAGCTGTGGCATCAAGTACTACAA | GATACCTGCCCTCCTTTCGTG |
| SNRPN | CCACCAGGCATTAGAGGTCC | AACAGTATGCTAAGGTCTTGGTGGA |
| NDN | GGTTTCTGACCCTCACCAGG | CTATAATGACCCACCCCCACC |
| GABRA5 | CCCAGCCAGGACAGTTTTTG | AGGGTCGTCATGGTCAGCA |
| GABRB3 | AATGTTGTCTTCGCCACAGGT | CCGAAAGCTCAGTGACAGTCG |
| GABRG3 | GCTACGCCAGCAAGAACAGC | GGTCATGGTCAGCACCGTG |
| GABRA2 | TATGGAATATACAATTGATGTTTTCTTTCG | CGTTCATCTTTCCATTTTTGTCG |
| GABRG1 | CAGAAAGCTAAAAAATAAAGCCTCG | GATCCAGGATGGAGACCAGGA |
